# Case Report: Acute abdomen in pregnancy—from appendicitis to diagnosed omental infarction

**DOI:** 10.3389/fsurg.2026.1799105

**Published:** 2026-05-01

**Authors:** Xiqiang Zhuang

**Affiliations:** Department of General Surgery, The Second Affiliated Hospital of Fujian Medical University, Quanzhou, China

**Keywords:** acute abdomen in pregnancy, acute appendicitis, case report, diagnostic laparoscopy, omental infarction in pregnancy

## Abstract

**Background:**

Omental infarction in pregnancy (OIP) is rare. Its symptoms, clinical signs, and imaging features are frequently non-specific, posing tremendous diagnostic challenges that are easy to overlook and may lead to severe implications. Improving doctors' understanding of omental infarction facilitates early detection and decreases the likelihood of severe consequences, including death.

**Case summary:**

We report a case of OIP presenting with right lower abdominal pain that was misdiagnosed as acute appendicitis. The patient underwent a laparoscopic omentectomy and appendectomy. This case aims to help clinicians better understand OIP and reduce the severe consequences of delayed diagnosis. Through timely surgical exploration, physicians can achieve early identification of OIP and initiate surgical intervention, thereby decreasing the devastating repercussions of a delayed diagnosis.

**Conclusion:**

This article aims to improve clinicians' understanding of OIP and propose a method for the diagnosis and treatment of OIP, while underscoring the value of diagnostic laparoscopy in establishing a definitive diagnosis when non-invasive modalities are inconclusive.

## Introduction

Here, we report a case of acute abdomen in pregnancy (AAP), which was initially misdiagnosed as appendicitis and later confirmed by surgery to be omental infarction in pregnancy (OIP). Finally, the patient underwent laparoscopic omentectomy and appendectomy and was discharged on day 3 post-operation. This research aims to improve doctors' awareness of OIP in diagnosing and treating AAP and present diagnostic thinking to improve the early detection rate and lower the risk of serious consequences, including death, for both the mother and the fetus. When the non-invasive evaluation is inconclusive, diagnostic laparoscopy offers a safe and definitive solution.

## Case presentation

### Chief complaints

The patient complained of right lower quadrant pain for 1 day with acute exacerbation over 3 h.

### History of present illness

**Figure 1:**
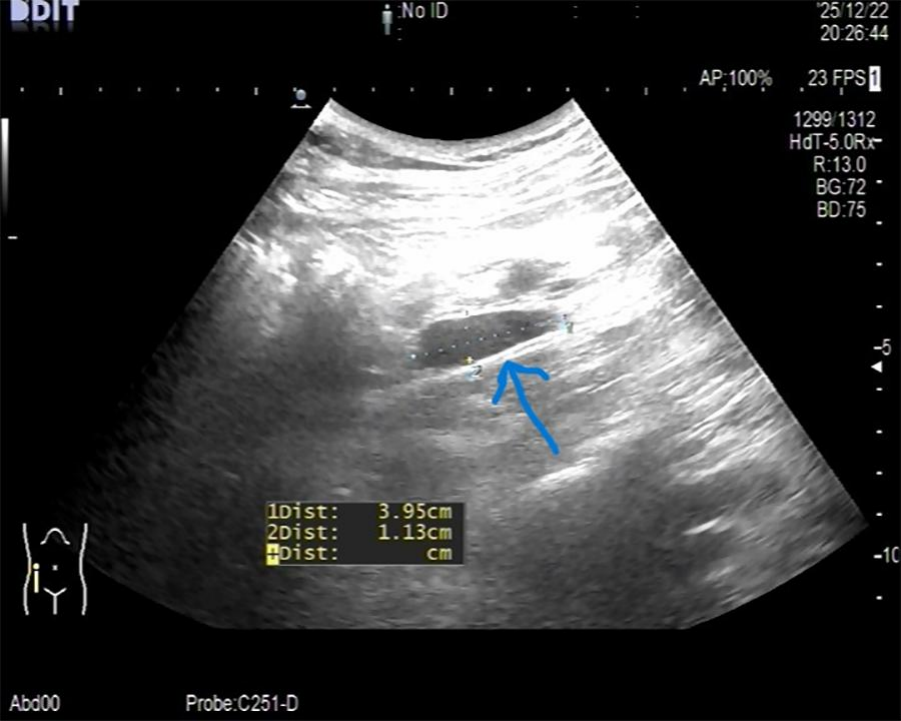
Appendix ultrasound.

A woman at 18 weeks of gestation presented with a 1-day history of moderate, persistent, non-radiating right lower quadrant pain. The pain acutely worsened 3 h prior without change in character, and was not associated with fever, chills, nausea, vomiting, diarrhea, hematuria, or dysuria. While obstetric, urinary, and appendiceal ultrasounds at a local hospital were unremarkable, a repeat ultrasound at our emergency department revealed a tubular hyperechoic structure in the right lower quadrant, suggestive of possible appendicitis (Figure 1).

### History of past illness

The patient had a history of tubal ligation in 2013, tubal recanalizations in 2021 and 2023, and a laparoscopic right salpingectomy in May 2025 due to an ectopic pregnancy.

### Personal and family history

The patient had no special personal or family history.

### Physical examination

The uterine fundus was 2 cm below the umbilicus. Right lower quadrant tenderness with rebound pain was noted, maximal at McBurney's point. No other abnormalities were detected.

### Laboratory examinations

The results of the laboratory tests were as follows: white blood cell count (WBC) of 11.15 × 10^9^/L and neutrophil (NE) of 9.26 × 10^9^/L.

### Imaging examinations

A tubular hyperechoic structure was visible in the right lower quadrant, initially suggestive of appendicitis. In retrospect, and in light of intraoperative findings confirming omental infarction (OI) with a normal appendix, this structure likely represented the infarcted omentum, highlighting a potential diagnostic pitfall in differentiating these conditions on ultrasound.

## Final diagnosis

Through laparoscopic examination, we ultimately determined that the patient had an OIP.

## Treatment

Due to the patient's persistent right lower quadrant pain and strong clinical suspicion for appendicitis, surgery was performed at the request of the patient and family. Intraoperatively, the appendix was directly visualized and appeared grossly normal: there was no significant dilation, thickening, hyperemia, exudate, or any signs of acute inflammation; However, a segment of the omentum in the right lower quadrant was dark, covered with purulent exudate, and adherent to the abdominal wall and uterus, leading to a diagnosis of OI. After discussing the intraoperative findings with the family, and explaining the diagnostic uncertainty, the potential benefits of appendectomy for ruling out future appendicitis, and the minimal additional risk, the family concurred with the surgical team's recommendation to proceed with concurrent appendectomy. Ultimately, laparoscopic omentectomy and appendectomy were performed.

**Figure 2:**
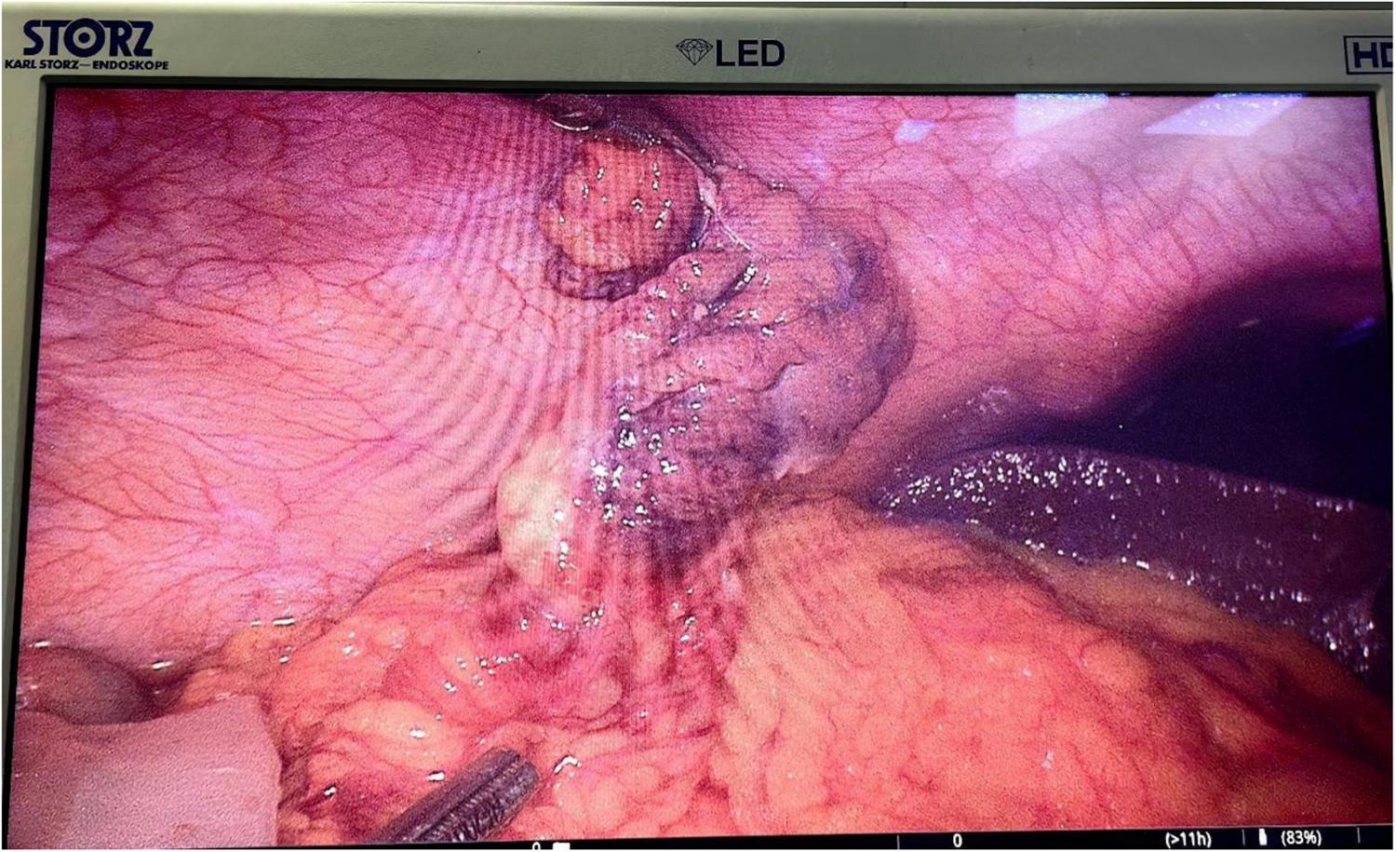
Intraoperative exploration of the omentum.

Due to the malfunctioning of the operating room video recording system, this image was captured by the anesthesiologist using a handheld device through the laparoscopic monitor, which accounts for the suboptimal quality. Despite this limitation, Figure 2 demonstrates a segment of the omentum in the right lower quadrant that appears dark and discolored. Intraoperatively, this segment was observed to be covered with purulent exudate and adherent to the abdominal wall and uterus, consistent with OI.

## Outcome and follow-up

After the operation, the patient was in good condition, and the right lower quadrant pain disappeared. The patient reported a little incision site pain on the first day after the procedure, but no further noticeable discomfort was noted and the follow-up blood test results were as follows: WBC of 7.79 × 10^9^/L and NE of 6.54 × 10^9^/L. During the regular follow-up process of 1 month after surgery, the patient and fetus showed no abnormalities.

## Discussion

AAP occurs in approximately 1/635 to 1/500 pregnancies. Due to significant physiological and anatomical changes, clinical presentations are often atypical, and the value of imaging examinations such as CT is limited because of potential fetal risks. These factors contribute to diagnostic challenges in the early stages. AAP can progress rapidly and, in severe cases, threaten the lives of both the mother and the fetus. Early recognition relies on careful clinical assessment and targeted auxiliary examinations.

Acute appendicitis is the most common surgical cause of AAP and can occur at any gestational stage. Without perforation, the fetal loss rate is 3%–5%; however, following perforation, fetal loss increases to approximately 36% (1), with a maternal mortality rate of approximately 4% (2). Therefore, acute appendicitis in pregnancy requires prompt management and vigilance against perforation. As the uterus enlarges, the appendix shifts upward. This begins around 12 weeks and reaches the level of the iliac crest by 24 weeks (3). In the mid to late trimesters, the enlarged uterus elevates the abdominal wall peritoneum, often obscuring classic signs such as tenderness, rebound pain, and muscular rigidity, with right lower quadrant pain the most common symptom (4).

The patient was admitted with right lower abdominal pain. Tubal pregnancies occur more frequently in the right fallopian tube (5). Ultrasound examination ruled out urinary or obstetric causes and revealed a tubular hyperechoic structure in the right lower abdomen. Inflammatory markers were elevated, and a physical examination demonstrated tenderness and rebound tenderness in the right lower quadrant. Based on these findings, acute appendicitis during pregnancy was considered the most likely diagnosis. Although magnetic resonance imaging could have been performed for further evaluation of appendiceal inflammation, the patient and her family opted for surgical intervention due to persistent pain and the potential risk of miscarriage associated with delayed treatment. Intraoperatively, the appendix appeared normal; however, a segment of the omentum in the right lower quadrant was dark, covered with purulent exudate, and adherent to the abdominal wall and uterus, leading to a diagnosis of OI. After discussing the findings with the family, who requested concurrent appendectomy, laparoscopic omentectomy and appendectomy were performed.

OI can be either primary or secondary (6). Primary omental infarction is rare, with about 400 cases reported in the literature (7). In the majority of cases, it is secondary to an identifiable cause such as hernia, vascular thrombosis, neoplasms, abdominal pelvic adhesion, and inflammatory conditions (8). Obesity is an important risk factor for developing OI (9, 10). Our patient was in a state of pregnancy-induced hypercoagulability, had gained weight, and had a history of prior abdominal surgery. These findings, combined with intraoperative adhesions, supported the diagnosis of secondary OI. Therefore, secondary OI was considered; pregnancy likely played a significant contributory role.

The hypercoagulable state of pregnancy, characterized by increased levels of clotting factors and decreased fibrinolysis, may have predisposed the patient to thrombotic events within the omental vasculature. These pregnancy-specific factors, combined with the patient's pre-existing adhesions, likely created a ‘perfect storm' that led to an OI. Therefore, while OI is rare, pregnancy may represent a unique risk period for its development, and clinicians should maintain a high index of suspicion when evaluating pregnant patients with AAP.

In the past, OI was diagnosed intraoperatively, but with new advances in imaging technology, it is becoming more easily detectable outside the surgical theater (7). Contrast-enhanced CT (CECT) of the abdomen is the imaging modality of choice to diagnose OI (8). CECT reveals a well-defined mass with heterogeneous density, accompanied by linear high-density opacities (11, 12). OI is most frequently represented by fat stranding adjacent to the bowel wall and, in particular, fat stranding that is disproportionate to the degree of bowel wall thickening (7). In addition to CT, ultrasound may equally aid clinical decision-making, although it remains the second choice of imaging modality. On ultrasound, OI appears as an echogenic mass-like lesion (13). Moreover, ultrasound may misdiagnose OI as appendicitis. As CT was not performed due to pregnancy in our case, the initial ultrasound examination failed to identify an echogenic mass-like lesion, which, in this context, contributed to our initial admission diagnosis of appendicitis.

This case underscores the value of diagnostic laparoscopy when the preoperative evaluation is inconclusive. In our patient, despite clinical, laboratory, and ultrasound assessments, the diagnosis remained uncertain. Diagnostic laparoscopy served the following three critical functions: it provided definitive diagnosis (normal appendix and an infarcted omentum), enabled timely intervention, and avoided diagnostic delay with its potential fetal consequences. We advocate for the early consideration of diagnostic laparoscopy in pregnant patients with acute abdominal pain when the diagnosis remains uncertain after a standard evaluation.

The optimal management of OI remains debated. Conservative measures, including fasting and antibiotic therapy, may be employed (14, 15). While symptoms can resolve spontaneously in some patients, close monitoring is essential, and surgical exploration remains necessary if clinical deterioration occurs. Specifically, surgery is indicated if the abdominal pain progressively worsens within 24–48 h (16) or when the differentiation between an omental infarction and appendicitis is difficult (13, 17). A systematic review of 90 publications by Medina-Gallardo et al. (15) suggested that surgical intervention may shorten the length of hospital stay. Therefore, to establish a definitive diagnosis, alleviate symptoms, prevent abscess formation, and potentially reduce hospitalization time, we advocate for surgical exploration and treatment in cases of OI. This approach is also recommended for cases where differentiation from conditions such as appendicitis or intra-abdominal abscess is challenging. Laparoscopic excision is the procedure of choice in our case.

## Conclusion

OI should be included in the differential diagnoses while treating patients with AAP. We advocate for a proactive surgical approach in the management of OI diagnosed during pregnancy. Diagnostic laparoscopy is a valuable and definitive tool for establishing an accurate diagnosis.

## Data Availability

The original contributions presented in the study are included in the article/Supplementary Material, further inquiries can be directed to the corresponding author.
